# A Novel In Situ Self-Assembling Fabrication Method for Bacterial Cellulose-Electrospun Nanofiber Hybrid Structures

**DOI:** 10.3390/polym10070712

**Published:** 2018-06-28

**Authors:** Muhammad Awais Naeem, Pengfei Lv, Huimin Zhou, Tayyab Naveed, Qufu Wei

**Affiliations:** 1 Key Laboratory of Eco-Textiles, Ministry of Education, Jiangnan University, Wuxi 214122, China; mawaisnaeem@hotmail.com (M.A.N.); plyu@ncsu.edu (P.L.); 7160707013@vip.jiangnan.edu.cn (H.Z.); 2 College of Textiles, Donghua University, Songjiang District, Shanghai 201600, China; 414006@mail.dhu.edu.cn

**Keywords:** in situ self-assembly approach, bacterial cellulose, bio-polymers, electrospun nanofiber membranes (ENM), hydrophilicity, functional properties, hybrid mats

## Abstract

Self-assembling fabrication methodology has recently attracted attention for the production of bio-degradable polymer nanocomposites. In this research work, bacterial cellulose/electrospun nanofiber hybrid mats (BC/CA-ENM) were formed by incorporating cellulose acetate electrospun nanofiber membranes (CA-ENMs) in the fermentation media, followed by in situ self-assembly of bacterial cellulose (BC) nanofibers. ENMs exhibit excessive hydrophobicity, attributed to their high crystallinity and reorientation of hydrophobic groups at the air/solid interfaces. We aimed to improve the hydrophilic and other functional properties of ENMs. As-prepared nanohybrid structures were characterized using SEM and FTIR. SEM results revealed that in situ self-assembling of BC nanofibers onto the electrospun membrane’s surface and penetration into pores gradually increased with extended fermentation periods. The surface hydrophilicity and water absorption capacity of as-prepared hybrid mats was also tested and analyzed. Hybrid mats were observably more hydrophilic than an electrospun membrane and more hydrophobic compared to BC films. In addition, the incorporation of CA electrospun membranes in the culture media as a foundation for BC nanofiber growth resulted in improved tensile strength of the hybrid nanocomposites compared to ENMs. Overall, the results indicated the successful fabrication of nanocomposites through a novel approach, with samples demonstrating improved functional properties.

## 1. Introduction

Composite materials are normally obtained by combining polymers, fibers, and fillers, and usually, present better mechanical properties than the components alone. Recently, the development of sustainable biopolymers to replace petrochemical-based materials has attracted significant attention [1,2,3].

Cellulose acetate (CA) is a known ecofriendly and biodegradable regenerated cellulose material that can be easily fabricated as a semipermeable membrane, as well as fibers and films for textile, biomedical, and other applications [4,5]. BC is one of the strongest nanofibrous extracellular biodegradable polymer materials produced by nature, possessing high modulus and strength, estimated to be 114 GPa and in excess of 1500 MPa, respectively [6]. It is secreted by *Acetobacter xylinum* through a hierarchical cell-directed self-assembly process and is known to be a sustainable and promising nanofibrous material for various end uses. Unlike cellulose from plants, bacterial cellulose (BC) is chemically pure and free of lignin and hemicellulose, with fiber diameters ranging between 55 and 60 nanometers and unique characteristics [7,8]. Because of the high degree of polymerization, crystallinity, and ultrafine web-like fibrous network structure, BC fibers have higher tensile strength, stiffness, and higher thermal/chemical stability compared to conventional cellulosic fibers obtained from plants [7,9,10,11]. BC membranes obtained by means of static fermentation have highly porous structure and strong biocompatibility [7,12,13,14,15] and exceptional environmental biodegradability [16], along with easily controllable size, shape, and thickness [7,17,18].

Many methods have been used to fabricate BC, BC-based hybrids, and nanocomposites, for applications including binding agents for fibers and other materials [19], high-quality paper [7], cosmetics, foods [8], speaker diaphragms, textiles and apparel [19], artificial skin and blood vessels [20,21] nanocomposite membranes [9,17,18,19], and others [22,23]. In situ self-assembly fabrication is a unique approach to produce BC-based nanocomposites and hybrid structures. The *A. xylinum* bacterial strain has been known to preferentially cultivate on some natural fibers and polymer surfaces when incorporated in a culture medium, as compared to growth in a pure medium. Some natural fibers and polymers (e.g., starch) can offer ideal substrates for the bacteria to grow on. Consequently, such hybrid structures and nanocomposites can be fabricated by incorporating natural fibers or polymers during the fermentation process. Numerous hydroxyl groups found on the surfaces of cellulosic fibers and polymeric substrates develop strong interactions with BC, attributed to hydrogen bonding [19,24,25,26,27].

In general, BC-based nanocomposites are produced, either via blending BC with a second phase once the nanofiber network structure has formed or by the addition of a second phase into the culture media during the formation of the nanofibrous matrix, to create a double network structure [28,29,30]. In the these approaches, BC can be regarded as a matrix reinforced with polymeric or mineral fillers, or as an additional reinforcement to another polymer [28]. In situ self-assembly, electrospinning, particle deposition, dispersion, and casting methods have been utilized to improve the physical and functional properties of BC nanocomposites [7]. Electrospinning is a versatile and simple technique used to fabricate submicron polymeric nanofibers with different diameters and varied morphologies. It enables nanofibers to form a highly porous mesh with controlled pore size, also referred to as electrospun nanofiber membranes (ENM). ENMs have unique and interesting features, such as high surface area to volume ratio, good permeability, large porosity, good mechanical properties, etc. [31]. Some researchers have reported the use of BC whiskers/nanowhiskers as a reinforcement for electrospun nanofibers, where BC nanowhiskers were introduced into solutions prior to electrospinning [32,33]. The use of an in situ self-assembly approach to fabricate nanofiber-based hybrid fibrous structures, by directly adding microfibrillated cellulose (MFC) and natural fibers to the fermentation (culture) medium, helped achieve better mechanical properties [34].

Nevertheless, significant research has not yet been reported on in situ self-assembled nanocomposites of BC-reinforced electrospun nanofiber mats. By taking advantage of the porous structure of electrospun nanofiber membranes and the large surface area-to volume ratio of nanofibers, it is likely that BC nanofibers grow among the pores and voids of ENMs. A recently reported in situ self-assembling technique [35] has certain limitations towards confined BC growth as the suspension level and smooth horizontal placement of ENM cannot be controlled after immersion in a fermentation solution. Due to the gradual growth of BC nanofibers, an electrospun membrane tends to sink towards the bottom of a container, further hindering the localized growth of BC nanofibers. This research work reports a novel in situ self-assembling approach to fabricate BC-based hybrid nanocomposites using an electrospun nanofiber membrane as the foundation for BC nanofibers, in order to achieve improved functional and mechanical properties.

## 2. Experimental Section

### 2.1. Materials and Methods

Cellulose acetate (CA, *M*_n_ ~ 30,000 g·mol^−1^, 39.8 wt % acetyl content), NaOH, dimethyl sulfoxide (DMSO), diethylamino ethyl chloride, tetrahydrofuran (THF), were purchased from Sigma-Aldrich (Shanghai, China). All reagents were used without further purification.

### 2.2. Preparation of Electrospun Nanofibrous Membranes (ENM)

A solution of 20 wt % CA in 1:1 (*w*/*w*) THF/DMSO with 0.1 wt % diethylamino ethyl chloride was first prepared at room temperature. The solution was then loaded into a 30-mL plastic syringe equipped with an 18-gauge 90° blunt end stainless-steel needle. The electrospinning setup included an ES30P (purchased from the Gamma High Voltage Research, Inc. (Ormond Beach, FL, USA)) high-voltage power supply and a cylindrical collection plate roller with a diameter of 25 cm. During electrospinning, a positive high voltage of 15 kV was applied to the needle, and the flow rate of 0.8 mL/h was maintained using a positive displacement syringe pump (purchased from KD Scientific Inc. (Holliston, MA, U.S.)). The CA nanofibers were collected as an overlaid membrane on the electrically grounded aluminum foil that covered the roller. The rotational speed of this roller/collector during electrospinning was set at 100 rpm. A heating lamp ZLD-2 (Zolee, Taizhou, China) was used to dry the nanofibrous membrane during electrospinning, and the membrane was further dried in a vacuum oven at 80 °C for 12 h after electrospinning. The collected CA nanofibrous membrane had a thickness of ~145 μm and a mass per unit area of ~35 g/m^2^.

### 2.3. Bacterial Cellulose Film Synthesis

To produce BC films, *Acetobacter xylinum* (*G. xylinus*) bacterial strain was used in Hestrin and Schramm medium (0.6% glucose, 0.8% Bacto Peptone, 2.5% yeast) [36], dissolved in distilled water. The initial pH value was set to 5.0 and maintained throughout the fermentation process. The crystallizing dishes (95 mm dia. 20 mm H) were incubated statically at 30 °C and the samples formed on the air-medium interface were harvested after a five-day incubation period. The synthesized cellulose pellicles were immersed in 0.1 M NaOH for 4 h at 80 °C [37]. Subsequently, the pretreated BC was further rinsed three times with deionized water (neutral pH) to remove all remaining microbial contaminants and obtain purified pellicles, followed by freezing and vacuum drying (freezing: 4 h and vacuum drying: 24 h). The average thickness of the dried BC films was ~124 μm.

### 2.4. Fabrication of BC/CA-ENM Hybrid Mats

**Figure 1:**
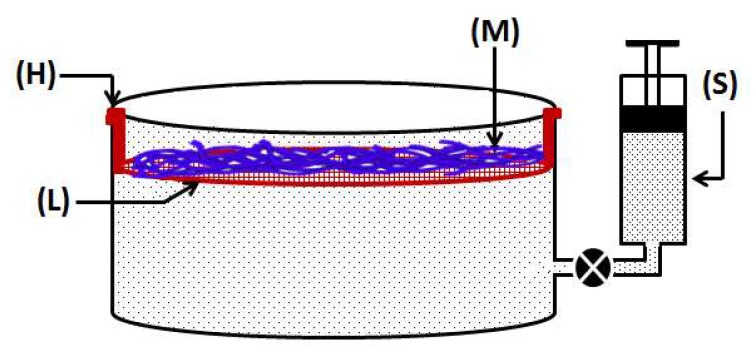
Schematic diagram of customized apparatus used for fabrication of in situ self-assembled bacterial cellulose-electrospun nanofiber hybrid mats.

In order to achieve confined growth of bacterial cellulose nanofibers, CA electrospun nanofiber membranes (CA-ENM) were used as a support. BC/CA-ENM hybrid mats were thus produced by means of an in situ self-assembly approach in a static incubator, containing the aforementioned culture medium. CA-ENMs were laid on a sterilized stainless steel thin wired lattice (L), which was securely placed inside glass container, and stabilized by supports (H), as shown in Figure 1. Afterward, the culture medium was injected into containers and inoculated using a BC producing strain, keeping the other parameters as reported earlier. Based on pre-experiments, the electrospun CA nanofiber membrane (M) was positioned slightly below (i.e., 1–2 mm) the surface of the culture medium throughout the fermentation period to allow the BC fibers to cover the top and grow freely between the openings and pores of ENMs. The size of the lattice and ENMs was the same as the surface area of the culture media. A specially customized microfluidics system (S) was connected to maintain the initial surface level and provide nutrients to the culture media as needed. The BC-modified mats were harvested after periods of three, five, and seven days to analyze the results of in situ self-assembly. After the complete incubation period (seven days), a layer of BC nanofibers was clearly observed to have self-assembled on the surface of CA-ENMs, forming a BC/CA-ENM hybrid structure. Hybrid mats were then purified, frozen, and vacuum dried following the same method described earlier. The average thickness of the dried hybrid mats was ~128 μm.

To ensure the homogeneity of the hybrid mats obtained, the central section (150 mm diameter) was used for analysis. Six samples each were prepared for: (1) pure bacterial cellulose (BC); and (2) BC/CA-ENM hybrid mats. Tests were performed on CA-ENMs, pure BC films, and hybrid mat samples to compare their mechanical and other functional properties.

## 3. Characterization

### 3.1. Scanning Electron Microscopy (SEM)

Surface and cross-section morphologies of specimens were studied using SU1510 Field Emission Scanning Electron Microscopy (Hitachi, Hitachi Ltd., Beijing, China) with accelerating voltage of 12.5 kV. First, bacterial cellulose and as prepared hybrid mats samples were oven-dried at 30 °C for 24 h to remove water. All samples were then coated with a 5 nm gold/palladium layer to enhance conductivity, and Image J 1.42q (developed by NIH, Bethesda, MD, USA) was used to evaluate nanofibers’ diameter.

### 3.2. Fourier Transform Infrared Spectroscopy (FTIR)

The BC, CA-ENMs, and BC/CA-ENM hybrid structures were characterized using an FTIR spectrophotometer (Thermo Scientific™ Nicolet™ iS™10, Thermo Electron Corporation, Waltham, MA, USA). ATR-FTIR spectra were taken in the range of 4000 cm^−1^ wavenumbers. Each scan was an average of 64 scans obtained at a resolution of 4 cm^−1^.

### 3.3. X-ray Diffraction Analysis (XRD)

The XRD analyses of BC film, CA membrane and BC/CA-ENM hybrid mats were done by using a diffractometer system (XRD; Bruker AXS, Karlsruhe, Germany). Scans were collected over the 2θ range of 20–80°, operating in the Bragg configuration using Cu-*K*α radiation (λ = 1.54 Å). Samples were first lyophilized by using a freeze dryer and then pressed into a thin layer (~1.0 mm) for analysis. 

### 3.4. Determination of Contact Angle

Contact angles were measured with the help of DCAT-21 (Data Physics Instruments GmbH, Tubingen, Germany) and SCA 20/21 software. The pure BC films and BC/CA-EMNs were individually mounted on glass slides using double-sided adhesive tape. Room-temperature deionized water (3 μL) was dropped onto the surface of samples using the sessile drop technique, and a photo was taken after the water droplet stabilized on the surface of the sample. The images were taken immediately after the drop landed on the surface of specimens. The corresponding contact angle was calculated by fitting the drop contour line numerically, using the Young-Laplace method. All contact angles were determined by averaging the values recorded at three different positions on each sample’s surface. The dynamic wettability was assessed by monitoring the contact angle change over time. For each sample, three videos recorded the variation in shape of drop during 25 s, taking one frame per second.

### 3.5. Water Absorption Capacity Analysis

Five specimens each for all types of samples were dried in air and weighed prior to immersion in DI water. After 48 h, excess unabsorbed water was removed and the specimens were weighed again. The water absorption capacity was estimated by the following equation:

*W* = (*W*_wet_ − *W*_o_)/*W*_o_,

where *W*_wet_ represents the weight of the wet specimen, and *W*_o_ represents the original weight of the dried specimen.

### 3.6. Mechanical Testing

Tensile strength of all samples was tested using universal tensile tester PT-119GDT (Baoda Instruments, Dongguan, China). A precise cutter severed the specimens into rectangular strips 10 mm wide and 50 mm long. Tensile strength test was conducted with a constant crosshead speed of 1 mm/min under ambient temperature and a humidity of 45% RH. The Young’s modulus values of all samples were determined from the tensile test results while a gauge length of 30 mm and a strain rate of 0.02/min were maintained using a 100 N load cell. Three parameters were recorded from each stress-strain curve: tensile strength, Young’s modulus, and elongation at break.

The modulus of elasticity (Young’s modulus) was calculated using the initial slope of the straight line of the stress-strain curve in the elastic region. Tensile strength was determined as the maximum point of the force-strain curve and mean values were computed from at least five separate tests for each sample type. Prior to testing, all specimens were conditioned at standard room temperature and humidity.

## 4. Results and Discussions

### 4.1. SEM Analysis

**Figure 2:**
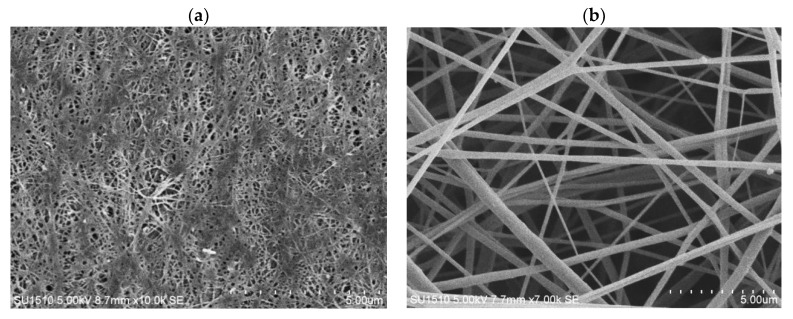
(**a**,**b**) SEM images for BC film (**a**) and CA-ENMs (**b**) sample surface morphology.

SEM analysis was carried out for BC film, CA membrane, and hybrid mats. The surface morphology of pure BC nanofibers and CA-ENMs is shown in Figure 2a,b.

As shown in Figure 2a, the surfaces of the BC consisted of a highly fibrous network-like structure consisting of ultrafine nanofibrils resembling randomly entangled ribbons. There are many hydroxyl groups in BC that exhibit a strong tendency to form intra- and inter-molecular hydrogen bonds. These cause the fibers to tightly entangle and form alternating bunches and clusters, resulting in uneven distribution [38].

As expected for CA-ENMs, Figure 2b shows an interconnective porous morphology attributed to electrospun membranes. Due to the porous structure of electrospun nanofiber membranes, BC nanofibers can easily grow among the pores to fill the openings and form nanofiber nets. The nanofibrous net structure was also reported by other researchers [39,40]. It is notable that the volume of BC nanofibers self-assembled (covering the surface and filling in the void openings) over electrospun nanofiber membranes increased over a prolonged cultivation period. BC nanofibers can also be seen in the vicinity of the interface with air, verifying a substantial surface coverage and penetration of BC nanofibers into the hybrid mats. The average fiber diameter of BC nanofibers and electrospun nanofibers was noted to be approx. 58–60 nm and 0.37 um, respectively.

**Figure 3:**
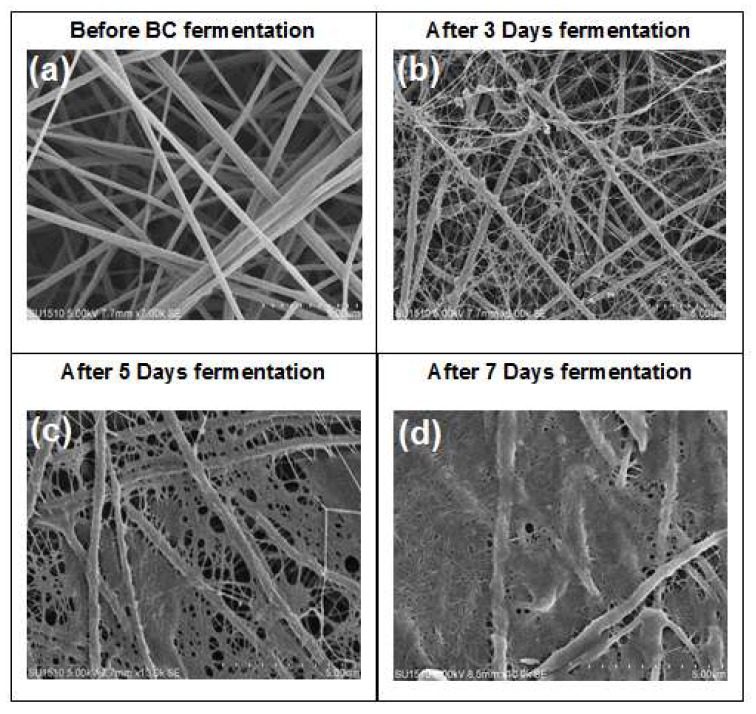
SEM images for BC/CA-ENM hybrid mats; surface morphology: (**a**) before fermentation; (**b**) after three days of fermentation; (**c**) after five days; and (**d**) after seven days.

**Figure 4:**
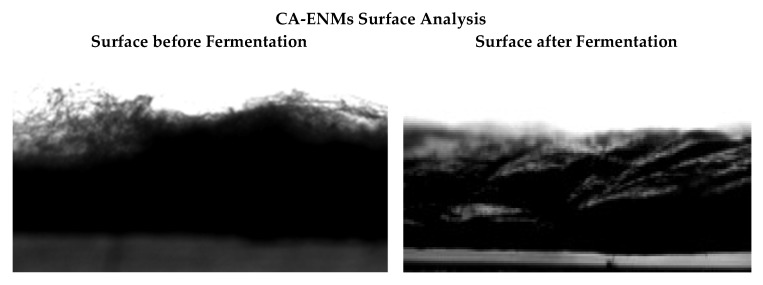
Photographs of CA-ENM surfaces before fermentation (**left**) and after seven days of fermentation (**right**).

The surface morphology of BC/CA-ENM hybrid mats is shown in Figure 3. SEM micrographs illustrate the periodic pattern of in situ self-assembly of BC nanofibers onto the electrospun membranes. It is notable that BC nanofibrous networks gradually started developing and largely filled up the openings and pores amongst electrospun CA nanofibers as the fermentation period extended. After seven days, the electrospun membrane can be seen to be almost fully covered by BC, exhibiting a smooth surface, free of rough fibers, as shown in Figure 4 (images taken using DCAT-21).

**Figure 5:**
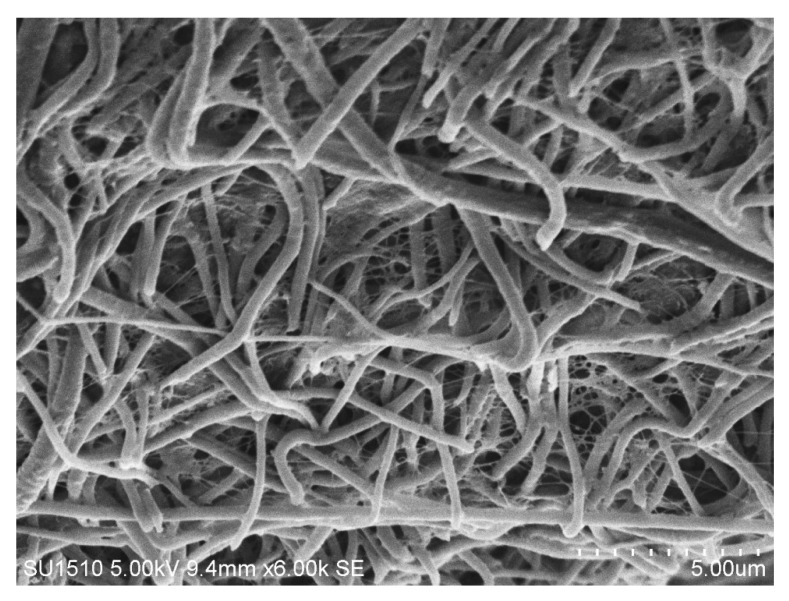
SEM images for BC/CA-ENM hybrid mats cross-sectional morphology.

The cross-sectional morphology of the BC/CA-ENM nanocomposites is shown in Figure 5. Growth of BC nanofibers can be noticed within pores/openings along the thickness of the electrospun hybrid mats. Such binding between CA electrospun fibers and BC nanofibers likely helped to achieve a well-integrated and compact structural arrangement and the resulting mechanical properties of nanocomposites.

### 4.2. FTIR Spectroscopy Analysis

**Figure 6:**
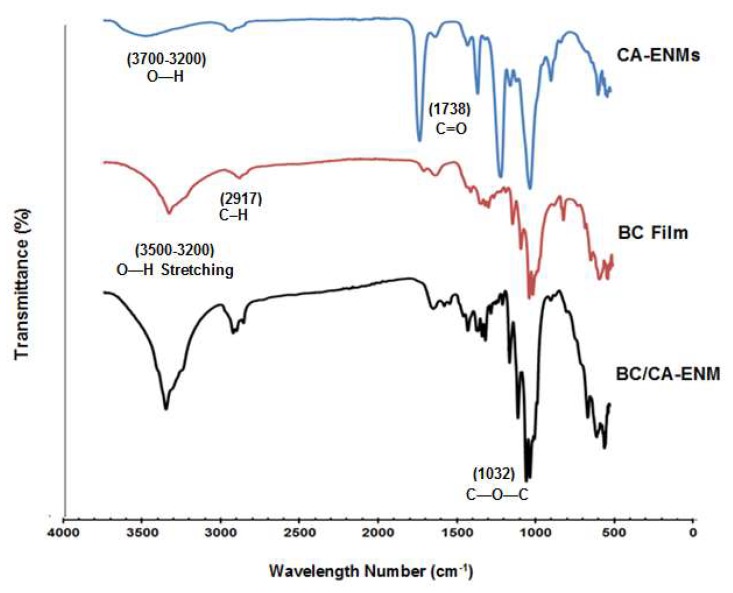
FTIR analysis graphs for BC films, CA-ENMs, and BC/CA-ENM hybrid mat samples.

Figure 6 shows the FTIR spectra of the pure BC film, CA-ENMs, and BC/CA-ENM hybrid mats. The broad band at 3200–3500 cm^−1^ that seen in the BC and BC/CA-ENM spectra is the result of the hydroxyl (O–H) stretching vibration resulting from the strong intra- and inter-molecular hydrogen bonds [38]. CA-ENM showed two strong adsorption bands at 1738 and 1219 cm^−1^, which are attributed to the C=O stretching and the acetyl groups, respectively. For BC film and BC/CA-ENM hybrid mats, characteristic peaks appeared at 2917 cm^−1^ (–CH_2_) and 3700–3200 cm^−1^ (–OH). According to He Jianxin [41], the adsorption band at 1640 cm^−1^ is assigned to the water adsorption, and 1362 and 1430 cm^−1^ to the symmetric and asymmetric vibrations of CH_3_. Other major absorption features appeared at 1686 cm^−1^ (–C=O), 1447 cm^−1^ (O=C–OR), 1365 cm^−1^ (–CH_2_), 1214 cm^−1^ (C–O), 1032 cm^−1^ (C–O–C), and 899 cm^−1^ (–CH). All specimen types exhibited similar characteristic features without introducing any new peaks. These results indicate that only physical blending occurred between the BC and CA nanofibers.

### 4.3. X-Ray Diffraction Analysis

**Figure 7:**
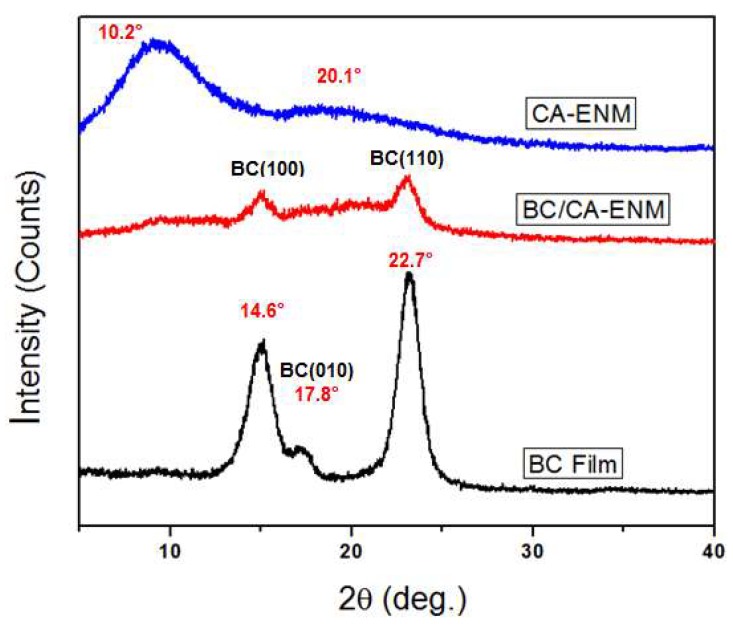
XRD diffractograms of BC films, CA-ENMs, and BC/CA-ENM hybrid mat samples.

BC is a semi-crystalline biomaterial that usually produces three major characteristic crystallinic peaks when analyzed through XRD. Figure 7 demonstrates XRD patterns of BC film, CA-ENM, and BC/CA-ENM samples. According to the Debeye-Scherrer equation [42], as below,

D=kλ/β cosθ,

where k is the shape factor, adopting a typical value of ~0.89, β is the breadth of the observed diffraction line at its half intensity maximum (FWHM), λ is the wavelength of X-ray source used in XRD, and θ corresponds to the peak position. The BC membrane exhibited three main peaks located at approximately 2θ = 14.6°, 17.8°, and 22.7°, corresponding to the crystallographic plane <110> <110> and <200> reflections, respectively, indicating the presence of cellulose I [43,44]. For CA-ENM, broad peaks appeared at approximately 10.2° and 20.1°, indicating an amorphous nanofibrous structure, which was consistent with He Jianxin et al. [16]. For BC/CA-ENM, the typical peaks observed at 14.6° and 22.7° are attributed to in situ self-assembled BC nanofibers; however, lesser intensities than those for the BC membrane were shown.

### 4.4. Wettability Analysis

**Figure 8:**
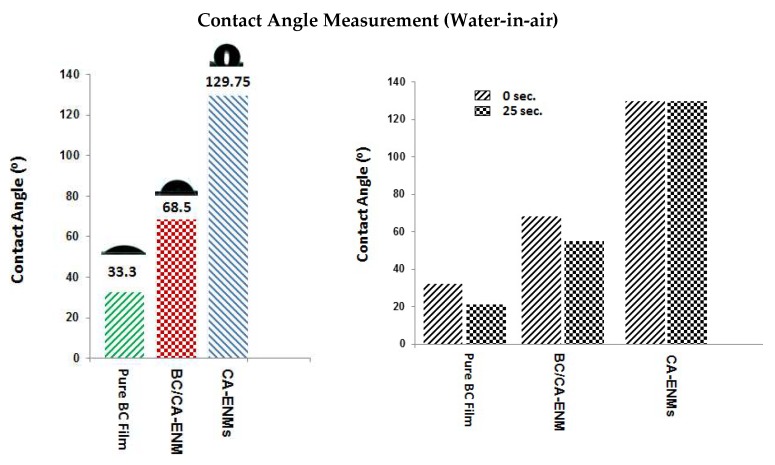
Contact angle measurement for pure BC films, BC/CA-ENM hybrid mats, and CA-ENMs.

The hydrophilic nature of bacterial cellulose identifies it as an excellent substrate for antibacterial, organic pollutant, and catalyst (improving the efficiency of enzyme loading) related applications. The static water contact angle could be regarded as a representative parameter to evaluate the surface hydrophilic or hydrophobic characteristics of nanocomposite membranes. Figure 8 shows the static water contact angle measurements used to compare the wettability of pure BC film, BC/CA-ENMs, and CA-ENMs. All three types of samples were subjected to a drop of deionized water.

The contact angle values obtained for pure BC, at 0 and 25 s of analysis (receding contact angle measurement using the Drop Length-Height method), were 33.30° and 22.12°, respectively. The same values determined for BC/CA-ENMs were 68.50° and 55.31°, respectively. Because of the surface roughness and very fine fiber diameters, electrospun membranes are known for their high surface hydrophobicity. CA-ENMs reported a contact angle of 129.75° and appear more hydrophobic than the other specimens. This phenomenon may be attributed to the rearrangement of CA molecules during the electrospinning process. Hydrophobic groups can get reoriented at the air/solid interface and hydrogen bonds might form in the interior membrane structure; similar findings were reported by Lin [45] regarding the PVA molecular rearrangement. Furthermore, the comparatively higher crystallinity of the electrospun fibrous membrane makes the diffusion and transformation of water molecules difficult, resulting in surface hydrophobicity [46]. It is interesting to note that surface hydrophilicity of BC/CA-ENM mats increased in comparison with CA-ENMs, which supports the spectrophotometry results that BC nanofibers largely cover the surfaces of hybrid mats.

Analysis of variance was performed to statistically analyze the wettability results, as shown in Table 1 and Table 2. It can be noticed that, at the level of 0.05, the data revealed there are significantly different in the means of the three types of samples.

### 4.5. Water Absorption Capacity (WAC)

**Figure 9:**
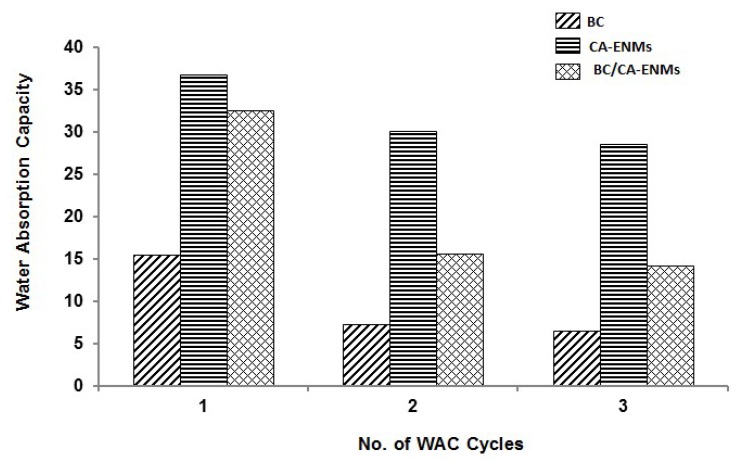
Percentage of the water absorbed in the BC films, CA-ENMs, and BC/CA-ENM hybrid mats after three wetting-drying cycles.

WAC quantifies the percentage of water absorbed in each nanostructure. All specimens were soaked in distilled water for 48 h and dried at room temperature to complete one cycle. The wetting and drying cycle was repeated twice and the results were recorded as shown in Figure 9. CA-ENMs demonstrate higher water uptake and retention, associated with their flexible and porous nanofibrous structure as well as their strong hydrogen bond interactions. However, after the in situ self-assembly of BC nanofibers, the surface of CA-ENMs might have become more compact, offering reduced porosity and inner spaces and hence minimizing the water uptake. Better WAC values shown by BC/CA-ENMs as compared to pure BC films can be assigned to the voids still present in the hybrid mat structure. This indicates that the hybrid structure achieved could offer a procedure to form nanofibrous structures that can absorb a relatively higher concentration of different compounds like enzymes, for improved activity and response.

Table 3 and Table 4 shows the statistical values and ANOVA test results. The results indicate that, at the level of 0.05, the data means of the three types of samples are significantly different.

### 4.6. Mechanical Properties

The mechanical properties of BC/CA-ENM hybrid mats were also tested in order to study the effect on tensile strength, breaking elongation, and Young’s modulus. Table 5 shows the mean value (μ) and standard deviation (σ) of the tested samples’ tensile properties (*n* = 5). The pure BC films and BC/CA-ENM hybrid mats show average tensile strength values of 3.42 MPa and 0.30 MPa, respectively, as shown in Table 1, whereas CA-ENMs displayed poor tensile strength (0.018 MPa) and a high elongation at break (33.58%).

Mechanical test results indicate that the in situ self-assembled BC nanofibers present inside BC/CA-ENM positively affect the tensile strength of the hybrid structure as compared to CA-ENMs. The Young’s modulus of the BC/CA-ENM hybrid mats was 0.97 MPa, which explains the stiffening of structure as compared to CA-ENMs reporting 0.05 MPa. We hypothesize that this improvement is because of new and additional links formed by BC nanofibers within the membrane structure during the self-assembling process. Table 5 indicates that, compared with pure CA-ENMs, the elongation of the BC/CA-ENM hybrid mats is slightly reduced (i.e., 1.9%), which might also be attributed to the comparatively stiff hybrid structure caused by BC nanofibers hindering the free sliding of CA electrospun fibers during elongation.

In addition, the tensile strength, elongation at break, and Young’s modulus were analyzed statistically. We performed an overall analysis of variance for tensile strength, elongation, and Young’s modulus, as illustrated in Table 6, Table 7 and Table 8 respectively. It can be noticed that, at the level of 0.05, the data also revealed a significant difference in the means of the three types of samples.

### 4.7. Flexibility Analysis

**Figure 10:**
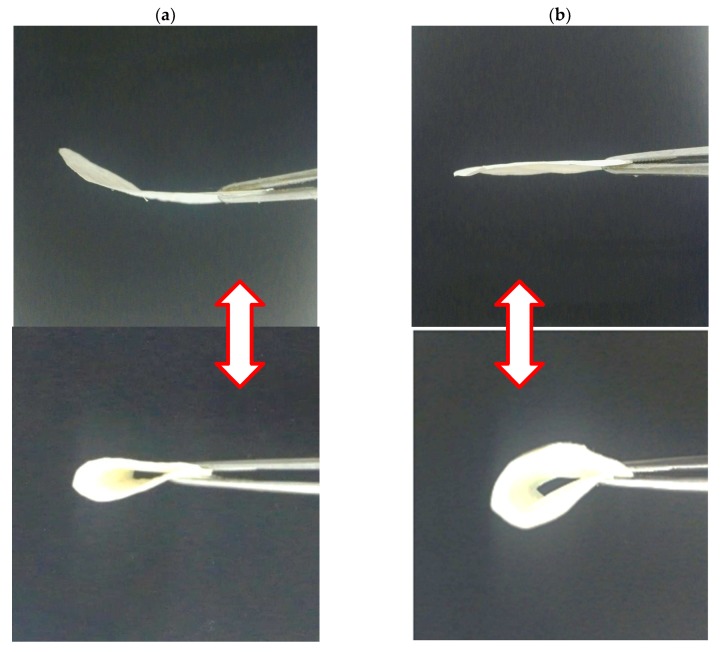
Flexibility analysis of Pure BC film (**a**) and as-prepared BC/CA-ENM hybrid mat (**b**) samples.

Flexibility is essential for high-strength membranes to be used in various applications and special fields [47]. Qualitative evaluation of flexibility behavior was performed by bending pure BC films and BC/CA-ENM mats using lab tweezers. Figure 10 shows that as-prepared hybrid mats could be easily bent and recovered, indicating improved elasticity over pure BC films. Hence, the current in situ self-assembling approach is also suitable to retain the flexibility of BC hybrid nanocomposite mats.

## 5. Conclusions

The in situ self-assembly method used herein for growing BC nanofibers onto electrospun nanofibers appears to be a promising approach that can help us to further investigate the prototyping possibilities in field of biosynthesis. XRD and SEM image provided evidence that *A. xylinum* can grow and produce BC on ENM. Mechanical testing results indicated that the incorporation of electrospun CA membranes in the BC culture media led to overall improved tensile properties of the BC-based hybrid structures compared to electrospun membranes. It is evident that binding within BC nanofibers and electrospun CA fibers contributed to improved surface and functional properties as well. The contained growth of BC has been demonstrated on the electrospun nanofiber membranes’ surface and, within ENM pores, it is possible and controllable during predetermined fermentation periods. Further study determining the prospects for using this in situ self-assembly methodology for growing multidimensional hybrid structures is needed.

## Figures and Tables

**Table 1:** Statistical results for water contact angle (advancing/reducing).

Sample type	Mean static contact angle (advancing)	Standard deviation (σ)	SE of mean	Mean static contact angle (Reducing)	Standard deviation (σ)	SE of mean
**BC film**	33.30	21.132	0.953	22.12	0.98039	0.43844
**CA-ENM**	129.75	1.356	0.606	128.61	1.24795	0.5581
**BC/CA-ENM**	68.51	0.619	0.277	55.30	1.24795	0.58662

**Table 2:** Overall analysis of variance for water contact angle.

Advancing contact angle						Reducing contact angle					
	DF	Sum of squares	Mean square	*F* value	Prob > *F*		DF	Sum of squares	Mean square	*F* value	Prob > *F*
**Model**	2	23,822.19161	11,911.09581	5278.07216	0	**Model**	2	29,694.81664	14,847.40832	10,507.3204	0
**Error**	12	27.08056	2.25671			**Error**	12	16.95664	1.41305		
**Total**	14	23,849.27217				**Total**	14	29,711.77329			

At the 0.05 level, the population means are significantly different.

**Table 3:** Statistical values for water absorbance capacity.

Sample type	Mean WAC (For 3 cycles)	Standard deviation (σ)	SE of mean
**BC Film**	29.07	0.843	0.377
**CA-ENM**	95.29	2.286	1.022
**BC/CA-ENM**	62.43	2.017	0.902

**Table 4:** Overall analysis of water absorption capacity.

	DF	Sum of squares	Mean square	*F* Value	Prob > *F*
**Model**	2	10,964.26389	5482.13195	1643.27567	2.33147 × 10^−15^
**Error**	12	40.0332	3.3361		
**Total**	14	11,004.29709			

At the 0.05 level, the population means are significantly different.

**Table 5:** Tensile test results for BC films and BC/CA-ENM hybrid mats and CA-ENMs.

Samples	Mean elongation at break (%)	Standard deviation (σ)	Mean tensile strength (MPa)	Standard deviation (σ)	Mean Young’s modulus (MPa)	Standard deviation (σ)
**BC films**	22.23	1.587	3.42	0.022	15.40	1.154
**BC/CA-ENM hybrid mats**	31.68	1.324	0.30	0.010	0.97	0.193
**CA-ENMs**	33.58	1.060	0.018	0.001	0.05	0.002

**Table 6:** Overall analysis of variance of tensile strength.

	DF	Sum of squares	Mean square	*F* value	Prob > *F*
**Model**	2	35.57463	17.78731	86,734.5389	0
**Error**	12	0.00246	2.05078 × 10^−4^		
**Total**	14	35.57709			

At the 0.05 level, the population means are significantly different.

**Table 7:** Overall analysis of variance of elongation.

	DF	Sum of squares	Mean square	*F* value	Prob > *F*
**Model**	2	369.9497	184.97485	102.76952	2.8175 × 10^−8^
**Error**	12	21.5988	1.7999		
**Total**	14	391.5485			

At the 0.05 level, the population means are significantly different.

**Table 8:** Overall analysis of variance of Young’s modulus.

	DF	Sum of squares	Mean square	*F* value	Prob > *F*
**Model**	2	736.80329	368.40165	726.97938	3.0087 × 10^−13^
**Error**	12	6.08108	0.50676		
**Total**	14	742.88437			

At the 0.05 level, the population means are significantly different.
